# Phylogeny and biogeography of South Chinese brown frogs (Ranidae, Anura)

**DOI:** 10.1371/journal.pone.0175113

**Published:** 2017-04-03

**Authors:** Yu Zhou, Sirui Wang, Hedan Zhu, Pipeng Li, Baotian Yang, Jianzhang Ma

**Affiliations:** 1 College of Wildlife Resources, Northeast Forestry University, Harbin, Heilongjiang, China; 2 Feline Research Center of the Chinese State Forestry Administration, Northeast Forestry University, Harbin, China; 3 College of Life Sciences, Shenyang Normal University, Shenyang, Liaoning, China; Sichuan University, CHINA

## Abstract

Few studies have explored the role of Cenozoic tectonic evolution in shaping the patterns and processes of extant animal distributions in and around East Asia. In this study, we selected South Chinese brown frogs as a model to examine the phylogenetic and biogeographical consequences of Miocene tectonic events within South China and its margins. We used mitochondrial and nuclear molecular data to reconstruct phylogenetic interrelationships among Chinese brown frogs using Bayesian and maximum likelihood analyses. The phylogeny results show that there are four main clades of Chinese brown frogs. Excepting the three commonly known Chinese brown frog species groups, *R*. *maoershanensis* forms an independent clade nearest to the *R*. *japonica* group. Phylogeny and P-distance analyses confirmed *R*. *maoershanensis* as a valid species. Among South Chinese brown frogs, there are four subclades associated with four geographical areas: (I) *R*. *maoershanensis*; (II) *R*. *japonica*; (III) *R*. *chaochiaoensis*; and (IV) other species of the *R*. *longicrus* species group. Divergence times, estimated using mitochondrial sequences, place the vicariance events among the four subclades in the middle to late Miocene epoch. Our results suggest that (1) South Chinese brown frogs originated due to a vicariance event separating them from the *R*. *chensinensis* species group at the time of the Geological movement (~18 million years ago, Ma) in southern Tibet and the Himalayan region; (2) the separation and speciation of *R*. *maoershanensis* from the *R*. *japonica* group occurred due to the dry climate at approximately 16 Ma; (3) South Chinese brown frogs migrated from South China to Japan at the time (~10.8 Ma) that the global sea-level fell and the East China Sea Shelf Basin was swamp facies, when a land gallery may have formed across the sea to connect the two areas; and (4) *R*. *chaochiaoensis* separated from other species of the *R*. *longicrus* species group during the uplift of the Tibetan Plateau at approximately 9.5 Ma.

## Introduction

The taxonomy of *Rana* (brown frogs), a genus of the family Ranidae, has been intensely debated in the last 20 years. *Rana* is widely distributed from the Western Palearctic to Northeast Asia. South China is one of the most richly diverse regions for brown frogs, with approximately 12 species [1, 2]. Most South Chinese brown frogs are classified in the *Rana longicrus* species group, which is one of the major species groups of Chinese brown frogs; the other two species groups are the *R*. *chensinensis* group and the *R*. *amurensis* species group [3, 4]. Additionally, the frogs of the *R*. *longicrus* species group were first known as the *R*. *japonica* group [5, 6] prior to their recognition as a new species and their classification into the *R*. *longicrus* species group based on morphological and nucleotide differences [4, 5].

In addition to the frogs of the *R*. *longicrus* species group, there are also four other commonly known species distributed in South China that share a close phylogenetic relationship with the three Chinese *Rana* species groups: *R*. *johnsi*, *R*. *shuchinae*, *R*. *zhengi* and *R*. *sauteri* [7]. Additionally, one endemic species, *R*. *maoershanensis* [8], is found only at the Maoershan National Nature Reserve in Guangxi Province in South China; its validity and phylogenetic relationships with other Chinese brown frogs remains unknown because of conflicting results in previous studies. *R*. *maoershanensis* was first reported as a new distribution record of *R*. *chaochiaoensis* from Guangxi Province [9] but was later described as a new species based on morphology [8]. Its status as a new species was supported by a phylogenetic analysis based on 16S ribosomal RNA [*16S*] showing that *R*. *maoershanensis* is closely related to the *R*. *chensinensis* species group [10]. Conversely, Yan et al. [11] considered *R*. *maoershanensis* as a junior synonym of *Rana hanluica* based on the mitochondrial cytochrome b [*Cytb*] and cytochrome coxidase subunit I [*COI*] genes. Therefore, substantial differences in the molecular phylogeny results of previous studies make *R*. *maoershanensis* a mysterious and controversial species. Therefore, a detailed estimate of the phylogenetic relationship of *R*. *maoershanensis* to other Chinese brown frogs from correct samples is necessary.

South Chinese brown frogs are common and widespread, from the southeast Chinese coastal hills to the Hengduan Mountains below 3,100 meters in elevation [4]. These species occur in a region of extreme Cenozoic tectonic and environmental changes [12], including the striking orogenesis of the Tibetan Plateau, which greatly altered the global climate [13–15]. Cenozoic global sea-level changes also affected the environment in East Asian coastal areas. Tectonic, environmental and sea-level changes could have affected the evolutionary history of amphibians because of their relatively low mobility, high philopatry, strict habitat specificity and physiological requirements [16]. In this study, we analyze both mitochondrial and nuclear genes to clarify the phylogeny and biogeography of frogs in South China, to reconstruct their phylogenetic relationships and to evaluate their evolutionary history.

## Materials and methods

### Sampling and laboratory work

**Fig 1:**
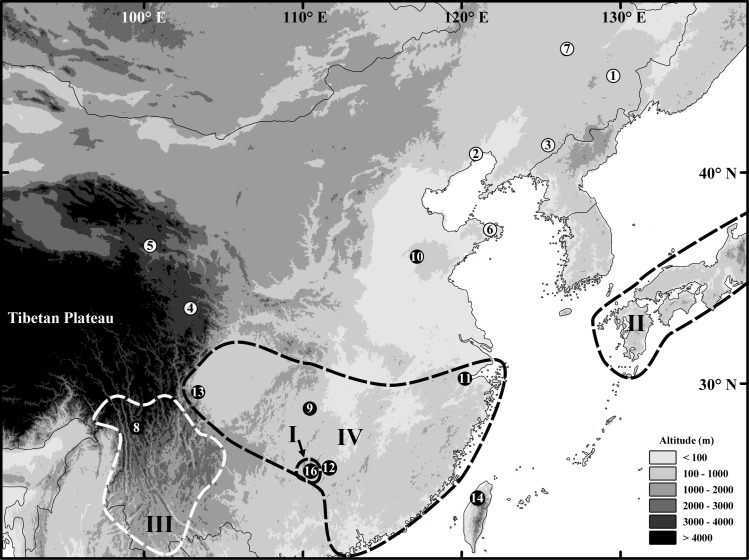
Sampling sites of *Rana* species used in this study. Numbers correspond with the species name list in Table 1. Contemporary distribution ranges of South Chinese brown frogs are divided into four clearly-defined areas within South China: I, Mount Maoershan; II, Japan; III, Middle to South Hengduan Mountains; and IV, low-altitude areas of South China. Elevation information was downloaded from STRM 90m Digital Elevation Data at http://srtm.csi.cgiar.org/.

We sampled 20 individuals of 15 *Rana* species, including two species of the *R*. *amurensis* species group, four species of the *R*. *chensinensis* species group and all nine ingroup species (the seven species of the *R*. *longicrus* species group, *R*. *japonica* and *R*. *maoershanensis*). This study included ten species that we sequenced ourselves and five species from GenBank; detailed information is shown in Table 1 and Fig 1. Within Table 1, The sequences with superscript numbers among the GenBank accession numbers are referenced as follows: 1, Zhou et al. [17]; 2, Che et al. [3]; 3; Zhou et al. [18]; 4, Yan et al. [11]; 5, Sumida et al. [19]; 6, Lin et al. [20]; 7, Ni et al. [21]. This study was conducted in strict accordance with the recommendations in the Guide for the Care and Use of Laboratory Animals of the National Institutes of Health. The protocol was approved by the Committee on Animal Care and the Ethics Committee of the College of Wildlife Resources, Northeast Forestry University (Permit Number: 100201–2015). For live frogs, we refer to Li et al. [22] only toe tip (1~2 mm^2^) tissues were collected and stored, and then they were released immediately after treating wounds with antiseptic. We have one week laboratory observation of *R*. *dybowskii* shown that the non-destructive sampling of toe tips minimizes the suffering of them and does not influence their survival. Toe tips were preserved in 95% ethanol.

**Table 1:** Information about brown frogs included in this study.

Species name	Voucher No.	Locality	Longitude	Latitude	Locality No.	GenBank accession numbers					
						12S	16S	Cytb	CO1	RAG2	LIG4
*R*. dybowskii*	SYNU08100690	Mudanjiang, Heilongjiang, China	129°33'	44°35'	1	KF204617	KF020592	KF020621	KF020606	KX025047	KX024980
*R*. chensinensis*	SYNU-hld1	Huludao, Liaoning prov., China	120°53'	40°45'	2	KF204616	KF020598^1^	KF020627^1^	KF020612^1^	KJ371973^1^	KJ371956^1^
*R*. huanrensis*	SYNU07040035	Huanran, Liaoning, China	125°25'	41°17'	3	KF204614	KF204642	KF204668	KX139725	KX139740	KX139734
*R*. *kukunoris*	SCUM045101WD	Luoergai Co., Aba State, Sichuan prov., China	102°56′	33°35′	4	DQ289091^2^	DQ289116^2^	JN984231^3^		-	-
	CJ06102001	Qinghai Lake, Qinghai prov., China	100°02′	36°37′	5			JN984213^4^	JF939073^4^		
*R*. coreana*	SYNU13030005	Mt. Kunyushan, Shandong prov., China	121°52'	37°12'	6	KX139718	KJ371937^1^	KJ371945^1^	KJ371941^1^	KJ371980^1^	KJ371965^1^
*R*. amurensis*	SYNU11100267	Taiyangdao, Heilongjiang prov., China	126°35'	45°47'	7	KX139717	KF020589^1^	KF020618^1^	KF020603^1^	KJ371975^1^	KJ371958^1^
*R*. *chaochiaoensis*	SCUM045096WD	Zhongdian, Yunnan prov., China	-	-	8	DQ289081^2^	DQ289106^2^			-	-
*R*. *jiemuxiensis*	KIZ05263	Jiemuxi National Nature Reserve, Hunan prov., China	110°24'	28°52'	9	-	-	JF939127^4^	JF939090^4^	-	-
*R*. culaiensis*	SYNU07050129	Mt. Culaishan, Shandong prov., China	117°21'	36°03'	10	KF204588	KF204620	KF204644	KX139726	KX139741	KX139735
*R*. zhenhaiensis*	SYNU08040100	Hangzhou, Zhejiang prov., China	120°08'	30°13'	11	KF204594	KF020599	KF020628	KF020613	KJ371970	KJ371954
*R*. hanluica*	SYNU07100490	Mt. Yangmingshan, Hunan prov., China	111°54'	26°06'	12	KF204607	HQ228158	KF020629	KF020614	KJ371968	KJ371949
*R*. omeimontis*	SYNU08060317	Mt. Emei, Sichuan prov., China	103°20'	29°29'	13	KF204605	KF204637	KF204661	KX139727	KX139742	KX139736
*R*. *longicrus*	long.T	Taipei, Taiwan prov., China	-	-	14	AB058863^5^	AB058881^5^			-	-
	KIZ15026	Nanzhuang, Miaoli, Taiwan prov., China	-	-				JF939107^4^	JF939067^4^		
*R*. *japonica*	jap. J^H^	Hiroshima, Japan	-	-	15	AB058858^5^	AB058876^5^			-	-
	KIZYPX11775	Japan	'-	-				JF939138^4^	JF939101^4^		
*R*. maoershanensis*	SYNU08030061	Mt. Maoershan, Guangxi prov., China	110°24'	25°52'	16	KX139719	KX139722	KX139731	KX139728	KX139743	KX139737
	SYNU08030062	Mt. Maoershan, Guangxi prov., China	110°24'	25°52'	16	KX139720	KX139723	KX139732	KX139729	KX139744	KX139738
	SYNU08030068	Mt. Maoershan, Guangxi prov., China	110°24'	25°52'	16	KX139721	KX139724	KX139733	KX139730	KX139745	KX139739
*Lithobates catesbeiana*		Jinhua, Zhejiang, China	-	-		KF049927^6^	KF049927^6^	KF049927^6^	KF049927^6^	-	-
*Lithobates sylvatica*		Southeastern Ontario, Canada	-	-		KP222281^7^	KP222281^7^	KP222281^7^	KP222281^7^	-	-
Babina daunchina*	SYNU12050567	Hangzhou, Zhejiang, China	120°08'	30°13'		KF204618	KF020600^1^	KF020630^1^	KF020615^1^	KJ371971^1^	KJ371955^1^

Table legend: Species names marked by asterisks were sequenced in our own laboratory. Numbers in Locality No. correspond to the collection locations in Fig 1.

Genomic DNA was extracted using a standard phenol-chloroform protocol [23]. The six gene fragments that we sequenced included four partial mitochondrial DNA (mtDNA) genes, namely, the 12S ribosomal RNA gene [*12S*], the 16S, the *Cytb*, and the *COI*, and two partial nuclear DNA (nuDNA) genes, namely, recombination-activating protein 2 [*RAG2*] and ATP-dependent DNA ligase IV [*LIG4*]. We amplified genomic DNA via the polymerase chain reaction (PCR) in 25-μl reactions using marker-specific primers (Table 2). Thermal cycling began with a denaturation period of 5 min at 95°C that was followed by 36 cycles of 94°C for 1 min, primer-specific annealing at 46–55°C for 2 min, and 72°C for 1 min, with a final extension at 72°C for 10 min.

**Table 2:** Primers used for PCR and sequencing.

Locus	Primer name	Primer sequence (5'-3')	AT	PS	Cited source
*12S*	FS01	AACGCGAAGATGAACCCAAAAAGTTCT	54	390	Sumida et al. [24]
	R16	ATAGTGGGGTATCATATCCCAGTTTGTTTT			
*16S*	F51	CCCGCCTGTTTACCAAAAACA	55	510	Sumida et al. [25]
	R51	GGTCTGAACTCAGATCACGTA			
*Cytb*	Cytba	AAGAAGATTTTGGCGATGGG	50	800	Zhou et al. [18]
	Cytbs	TAAATCTCACCCCCTCCTCAA			
	L14850	TCTCATCCTGATGAAACTTTGGCTC		570	Tanaka-Ueno et al. [26]
	H15502	GGATTAGCTGGTGTGAAATTGTCTGGG			
*CO1*	Chmf4	TYTCWACWAAYCAYAAAGAYATCGG	50	650	Che et al. [27]
	Chmr4	ACYTCRGGRTGRCCRAAR AATCA			
	L-turtCOIc	TACCTGTGATTTTAACCCGTTGAT		800	Stuart and Parham [28]
	H-turtCOIc	TGGTGGGCTCATACAATAAAGC			
*RAG2*	RAG2_F1	TTWGGNCARAARGGNTGGCCNAA	46	800	Shen et al. [29]
	RAG2_R1	CATRCAYTGNGCRTGNACCCARTG			
	RAG2_F2	AGGGTTTTCCCAGTCACGACGGWGGKAA RACNCCNAAYAAYGA			
	RAG2_R2	AGATAACAATTTCACACAGGCARCAYTTD ATCCARTANCC			
*LIG4*	LIG4_F1	GAYTCNTTYTAYCCNGCNATG	46	1000	Shen et al. [29]
	LIG4_R1	TCMGGYTTDATYTTNARCCANCC			
	LIG4_F2	AGGGTTTTCCCAGTCACGACAGRATGGCB TAYGGMATHAARGA			
	LIG4_R2	AGATAACAATTTCACACAGGGTTCMCCDC KTTTRTCYGGYTTGTA			

Abbreviations: AT, annealing temperature (°C); PS, approximate product size (bp).

### Sequence analyses

Nucleotide sequences were edited using MEGA 7 [30] and aligned using the L-INS-i method in MAFFT version 5 [31] with the default parameters. Ambiguous alignments were removed using Gblocks ver. 0.91b [32] with the ‘with half’ option and the default block parameters. Mitochondrial (Mt) and nuclear genes from *Babina daunchina*, which we sequenced ourselves, and Mt genome sequences of *Lithobates* (*L*. *catesbeiana* and *L*. *sylvatica*) from GenBank [20, 21] were selected as outgroups for phylogeny and divergent time analyses. P-distances were calculated using MEGA v.7.0. *B*. *daunchina*, *L*. *catesbeiana* and *L*. *sylvatica* were selected as outgroup taxa based on the results of Frost et al. [2].

Partitioned Bayesian (BA) and maximum likelihood (ML) analyses were performed on the concatenated mtDNA and concatenated mtDNA+nuDNA datasets. For mtDNA phylogenetic analyses, an 8-partition scheme was applied: the *12S* and *16S* genes were treated as two separate partitions, and *Cytb* and *COI* were partitioned according to codon positions. For mtDNA+nuDNA, six additional partitions were added relative to the mtDNA phylogeny analyses, with a 12-partition scheme according to the codon positions of *RAG2* and *LIG4*. The incongruence length difference test (ILD) [33] was used to assess the informational congruence between mtDNA and nuDNA and was performed with PAUP version 4.0b10 [34].

For BA analyses, each partition had independent models of substitution as suggested by MrModeltest v.2.3 [35] using the Akaike Information Criterion (Table 3). Markov chain Monte Carlo (MCMC) was run for 10 million generations and was implemented in MrBayes v.3.1.2 [36]. Trees were sampled every 1,000 generations. Stationarity was checked graphically by plotting log-likelihood scores in Tracer ver. 1.6 [37]. The first one million generations were discarded as burn-in, and the remaining trees were used to build a consensus tree.

**Table 3:** Nucleotide substitution models selected in MrModeltest using the Akaike Information Criterion (AIC).

No. of partition	Gene	Partition	AIC model
1	*12S*		GTR+G
2	*16S*		GTR+I+G
3	*Cytb*	codon 1	K80+I+G
4		codon 2	HKY+I
5		codon 3	GTR+G
6	*COI*	codon 1	GTR+G
7		codon 2	HKY
8		codon 3	GTR+G
9	*RAG2*	codon 1	F81+I
10		codon 2	F81+I
11		codon 3	K80+I
12	*LIG4*	codon 1	F81+I
13		codon 2	HKY
14		codon 3	HKY+G

The ML analyses was implemented using a rapid-hill-climbing algorithm in RAxML v.7.0.4 [38]. First, the best-scoring ML tree was inferred with 200 replicates under the CTRCAT model. Next, a nonparametric bootstrap analysis with 200 replicates was conducted under the CTRCAT model.

### Divergence time estimates

The estimation of divergence time was performed in BEAST ver. 1.8.3 [39]. We assumed a relaxed lognormal clock (uncorrelated) for the rate-variation model and a Yule process for the tree prior. An independent substitution model was assigned to each partition corresponding to the BI models. One constraint, with a calculated age of 31.2 ± 8.1 million years ago (Ma) and representing the split between *Rana* and *Lithobates* and corresponding to node 19 in the study by Bossuyt et al. [40], was imposed on the tree to establish the divergence time. Analyses were undertaken with 20 million generations while sampling every 1,000th tree; the first 25% of sampled trees were treated as burn-in. Burn-in and convergence of the chains were determined with Tracer ver. 1.6 [37].

## Results

### Sequence characteristics

A total of ~4,200 sites were obtained, among which ~2,410 sites were from mtDNA and ~1,800 sites were from nuDNA. All sequences were deposited in GenBank; detailed information is shown in Table 1. From mtDNA, all species except *R*. *jiemuiensis* had sequences for all four mtDNA loci. For twelve species, the mtDNA sequences of each species were from the same specimen. For the remaining three species, *R*. *kukunoris*, *R*. *longicrus* and *R*. *japonica*, the mtDNA sequences were from different sources and were concatenated together based on direct or indirect evidence to represent the genetic characteristics of one species. A detailed description of the direct or indirect evidence for concatenating sequences from different sources is described in S1 Text. In the nuDNA dataset, ten species had both nuDNA genes (Table 1). The results of the ILD test showed no significant phylogenetic incongruence (P = 0.985) between the mtDNA and the nuDNA; therefore, we combined them into a single dataset for the phylogeny analyses.

### Phylogeny, nucleotide diversity and divergence times

**Fig 2:**
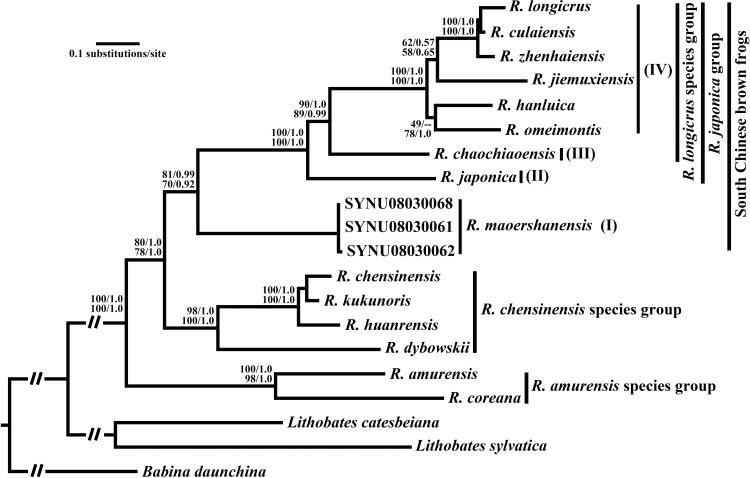
Topology of maximum-likelihood analysis based on combined data from mitochondrial genes. The numbers along the branches are the support values for the maximum-likelihood inference (ML) and Bayesian posterior probability (BPP) and are shown as ML/BPP by the combined data from mitochondrial genes at the upper area of each branch number and as ML/BPP by the combined data from mitochondrial genes and nuclear genes at the lower area of each branch number.

BA and ML analyses of the mtDNA dataset and the mtDNA+nuDNA dataset inferred similar stable topologies, with four well-supported clades of Chinese brown frogs (Fig 2). Three clades corresponded to previously recognized species groups, namely, *R*. *amurensis*, *R*. *chensiensis*, and *R*. *longicrus* (or *R*. *japonica*), while the fourth clade represented *R*. *maoershanensis*. Phylogenetic analyses showed that *R*. *maoershanensis* had a close relationship with the *R*. *longicrus* species group, with strong support [ML bootstrap (ML) = 81% and Bayesian posterior probability (BPP) = 0.99 by mtDNA; ML = 70% and BP = 0.92 by mtDNA+nuDNA]; together, they displayed a sister relationship with the *R*. *chensiensis* species group.

There were four main conspicuous subclades within our ingroup species, three of which are South Chinese subclades, namely, *R*. *maoershanensis* (subclade I), *R*. *chaochiaoensis* (III) and other species in the *R*. *longicrus* species group (IV), and one of which is a Japanese subclade with *R*. *japonica* (II). Within the *R*. *japonica* group, subclades III and IV have the closest relationship, and they then join with subclade II, all with absolute supports.

P-distances between *R*. *maoershanensis* and the other Chinese brown frogs were all above 13.2% for *Cytb* gene sequences and above 12.2% for *COI* gene sequences (S1 Table). Compared to the major clades, *R*. *maoershanensis* differed by at least 16.1% and 12.7% in the *Cytb* and *COI* genes, respectively (S2 Table).

**Fig 3:**
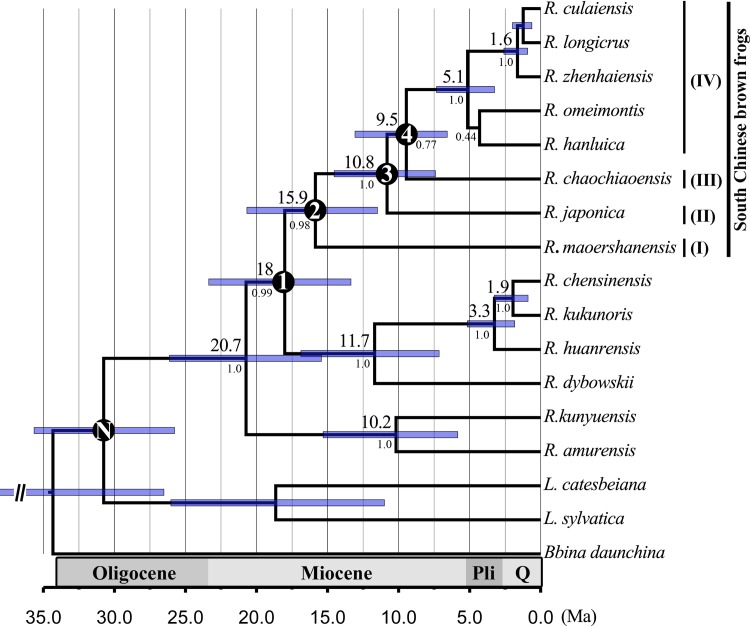
Estimates of divergence times obtained with BEAST 1.8.3. Values above each node show average ages (Ma), and values below each node show Bayesian Posterior probabilities by BEAST analysis. Bars show 95% highest posterior density (HPD) intervals. For black cell nodes, node N is the constraint point during molecular dating analyses. I-IV correspond to the subclade designation in Fig 2. 'Q' and 'Pli' are abbreviations for Quaternary and Pliocene, respectively.

The timing of our favored internal nodes of Chinese brown frogs is shown in Fig 3. The crown age of Chinese brown frogs is circa 20.7 Ma (95% highest posterior density (HPD) intervals, 15.4–26.1 Ma), which also represents the divergence time of the *R*. *amurensis* species group and other Chinese brown frogs. The split between the *R*. *chensinensis *species group and the South Chinese brown frog group occurred approximately 18 Ma (95% HPD: 13.4–23.4 Ma). The divergence time between *R*. *maoershanensis* and the *R*. *japonica* group is approximately 16 Ma (95% HPD: 11.5–20.7 Ma). The divergence time between *R*. *japonica* and the *R*. *longicrus* species group is 10.8 Ma (95% HPD: 7.4–14.5 Ma), and that between *R*. *chaochiaoensis* and other species of the *R*. *longicrus* species group is 9.5 Ma (95% HPD: 6.6–13.1 Ma).

## Discussion

### Phylogeny of South Chinese brown frogs

Our study provides a well-resolved phylogeny of Chinese brown frogs, with a near-complete taxonomic sampling of the three commonly known Chinese *Rana* species groups (13 of 14 species) and of one *R*. *longicrus* species group with a related species, *R*. *japonica*, from Japan. The South Chinese brown frogs first have a sister relationship with the *R*. *chensinensis* species group; these groups together have a sister relationship with the *R*. *amurensis* species group. Within South Chinese brown frogs, there are two main clades, namely, the *R*. *japonica* group and a single-species clade of *R*. *maoershanensis*. Within the *R*. *japonica* group, there are four subclades associated with four geographical areas. The subclades are *R*. *maoershanensis* (I); *R*. *japonica* (II); *R*. *chaochiaoensis* (III); and other brown frogs from the *R*. *longicrus* species group (IV).

## Validity of *R*. *maoershanensis*

Although previous studies have disagreed on the taxonomic status of *R*. *maoershanensis* [11], our phylogeny confirms the validity of this species. We performed phylogenetic analyses on independent genetic datasets (mtDNA and mtDNA+nuDNA), and all of our results recovered a monophyletic *R*. *maoershanensis* (Fig 2) that is distinct from the three other commonly known Chinese *Rana* species groups.

P-distances have been used as a line of evidence to delineate species among Chinese brown frogs [10, 41]. Kartavtsev et al. [42] used a large *Cytb* and *COI* dataset to compare genetic divergence at different levels of taxonomic rank and found that sibling species differed by P-distances of 5.52±1.34 (*Cytb*) and 4.91±0.83 (*COI*). Our values comparing *R*. *maoershanensis* with other Chinese brown frogs were above 0.13 for *Cytb* and above 0.12 for *COI*. Additionally, the P-distances between *R*. *maoershanensis* and other species groups were also larger than 0.16; all of these data indicate that *R*. *maoershanensis* differs at the species level from different genera within a family.

In recent studies sampling *Rana* species at Mount Maoershan and placing *R*. *maoershanensis* as a synonymous species with *R*. *hanluica* [11], we believe that incorrect samples were collected. Our samples were collected by the same *R*. *maoershanensis* finders as in Lu et al. [8]. The difficult sample collection of *R*. *maoershanensis* and the importance of its phylogenetic position also imply that this species needs more care and protection.

### Origin and biogeography of South Chinese brown frogs

**Fig 4:**
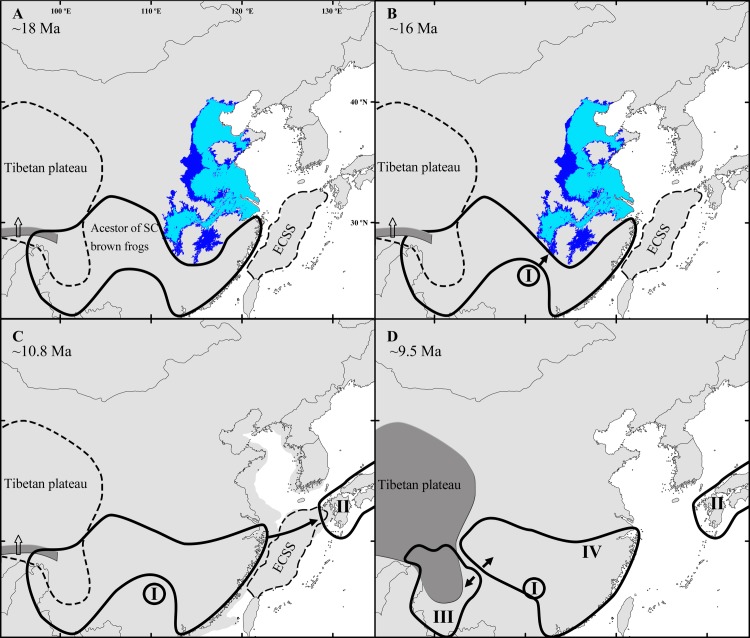
Sketch maps of evolutionary scenarios for South Chinese brown frogs. (A) Potential distribution of the South Chinese brown frog ancestor after the vicariance event with the *R*. *chensinensis* species group by the Geological movement that occurred in southern Tibet and the Himalayan region approximately 18 Ma. Dark blue and light blue represent areas with altitude <100 m and <50 m, respectively. (B) Clade I separation from other species of South Chinese brown frogs due to the dry climate at approximately 16 Ma. (C) Dispersal from Southeast China to the Japan Islands by a fall in the global sea level; the East China Sea Shelf Basin (ECSSB) was swamp facies at approximately 10.8 Ma, before vicariance between the two areas. (D) Vicariance between the Middle to South Hengduan Mountains (clade III) and other low-altitude regions (clade IV) by uplift of the Tibetan Plateau approximately 9.5 Ma. Clades are defined in Fig 2.

From our own and previously published *Rana* location data [4, 11, 18], we first conclude that the main boundary between the South Chinese brown frog and the *R*. *chensinensis* species group runs from the Sichuan basin to the middle and lower Yangtze River plain. *R*. *chaochiaoensis* from among the South Chinese brown frogs and *R*. *kukunoris* from the *R*. *chensinensis* species group have adjacent plateau distributions around Tibetan plateau and they split by middle Hengduan Mountains and Sichuan Basin. Based on our divergence-time analyses, the South Chinese brown frogs display a sister relationship with the *R*. *chensinensis* species group (Fig 2) and they diverged at 18 Ma (95% HPD: 13.4–23.4 Ma). Approximately 23–17 Ma, the global sea-level rose by over 100 m [43–45]; the sea-level rise could have caused most of the middle and lower Yangtze River plain to be flooded by seawater (Fig 1). Therefore, we argue that brown frogs could not have migrated across the lower Yangtze River plain before 17 Ma and that the origin of South Chinese brown frogs was not in these areas. During 18–8 Ma, the change in the Tibetan deformation pattern occurred when north-south contraction was replaced by coeval development of conjugate strike-slip faulting and east-west extension in Tibet [46–51], which may be the main reason that South Chinese brown frogs separated from the *R*. *chensinensis* species group. Our analyses suggest that the common ancestor of South Chinese brown frogs and the *R*. *chensinensis* species group was widely distributed throughout the Tibetan area before 18 Ma, after which a vicariance event of the two main clades, the South Chinese brown frog clade and the *R*. *chensinensis* species group clade, was caused by the Geological movement that occurred in southern Tibet and the Himalayan region (Fig 4A).

Currently, *R*. *maoershanensis* is found only at an approximately 2,000-m elevation on Mount Maoershan in Guangxi Province, China [8]. *R*. *maoershanensis* is distributed near the boundary area of the *R*. *longicrus* species group, with an overlap in distribution with *R*. *zhenhaiensis* and *R*. *hanluica* in central South China (see Fig 1). According to our phylogeny and divergence-time analyses, *R*. *maoershanensis* has a sister relationship with the *R*. *japonica* group as a result of a vicariance event at ~16 Ma. Numerous studies suggest that an intensification of climatic aridity occurred in central Asia during the middle Miocene (16–12 Ma), based on pollen records in this area [52–55]. We suggest that the dry periods during this stage made the brown frogs’ habitat shrink and resulted in the separation and speciation of *R*. *maoershanensis* from the *R*. *japonica* group (Fig 4B).

After *R*. *maoershanensis* split with the *R*. *japonica *group, *R*. *japonica* separated from the *R*. *longicrus* species group at ~10.8 Ma, which was at the end stage of the late-Middle-Miocene sharp global sea-level decrease [44]. The amplitude of the late Middle Miocene sea-level decrease was 45–68 m [56], and the shallow sea areas of East Asian margins could have become land during the sharp sea-level decrease. The East China Sea Shelf Basin (ECSSB) entered a new developmental stage, the neotectonic movement stage, after the Miocene [57]. Previous geophysical prospecting and drilling data have confirmed that the ECSSB was swamp facies during the early to middle Miocene and was in the transition phase during the Pliocene [58, 59]. Therefore, there was once a land gallery connecting Southeast China and South Japan during the sea-level decrease and when the ECSSB was in its swamp stage. Our hypothesis suggests that the ancestor of *R*. *japonica* migrated from South China, for instance, from the Fujian region of China, to Japan through the land gallery at ~10.8 Ma. Separation between the *R*. *japonica* and *R*. *longicrus* species groups then occurred when the land gallery was destroyed as the sea level recovered and the swamp facies of the ECSSB subsided (Fig 4C).

Orogenesis is responsible for driving speciation in many taxa via vicariance. In the orogenesis of the Tibetan plateau, the rapid uplift and unroofing of southern Tibet began at approximately 20 Ma; the further significant increases in the altitude of the Tibetan plateau are thought to have occurred approximately 10–8 Ma [60–62]. For several species, the uplift of the Tibetan plateau has been hypothesized as the driving force of vicariant speciation (e.g., Macey et al. [63]; Che et al. [64]). *R*. *chaochiaoensis* is the only living species among South Chinese brown frogs that lives on a plateau; these frogs are mainly distributed at the 1,150–3,500-m plateau in the south part of the Hengduan Mountains [4]. Our divergence-time analyses show that *R*. *chaochiaoensis* separated from other species of the *R*. *longicrus* species group at approximately 9.5 Ma, coincident with the end period of the Tibetan Plateau uplift, suggesting that the geological event resulted in the speciation event (Fig 4D).

### A summary

This study evaluated the phylogeny and evolutionary history of the frequently studied South Chinese brown frogs. We successfully recovered the phylogenetic relationships of South Chinese brown frogs and most Chinese brown frogs using mitochondrial and nuclear genes. The phylogenetic analyses also confirmed *R*. *maoershanensis* as a valid species composing an independent clade that is distinct from the other three commonly known Chinese *Rana* species groups. By comparing our divergence-time estimates with the ancient climatic and geological history of the distribution areas of South Chinese brown frogs, our findings suggest that the middle Miocene dry climate, the late Middle Miocene sea-level decrease, the neotectonic movement of the ECSSB and the Tibetan plateau uplift all played important roles in shaping the disjunctive distributions of South Chinese brown frogs.

Similar biogeographical processes might be found for other taxa that possess distribution ranges in the Tibetan plateau and East Asian margins and have evolutionary histories during the Miocene. Previous biogeographical studies of Tibetan amphibians [18, 63, 64] also agree that the late-Miocene uplift of the Tibetan plateau may have resulted in speciation processes. Miocene migrations from South China to Japan were also observed in phylogenetic and biogeographical analyses of giant flying squirrels [65] and salamanders [66]. We are the first authors to suggest that the late-Miocene sea-level fall and the swamp facies of the ECSSB may have built a land gallery for the migration of some organisms between South China and Japan.

## Supporting information

S1 Table P-distances among species of Chinese brown frogs based on *Cytb* (below the diagonal) and *COI* (above the diagonal). (DOCX)

S2 Table P-distances among four main clades of Chinese brown frogs based on *Cytb* (below the diagonal) and *COI* (above the diagonal). (DOCX)

S1 Text Combination of same-species sequences from different specimens. (DOCX)
