# Supplementary material for: Phylogeny and biogeography of South Chinese brown frogs (Ranidae, Anura)
Source: PLoS One. 2017 Apr 3;12(4):e0175113. doi: 10.1371/journal.pone.0175113 (PMC5378408; doi:10.1371/journal.pone.0175113)
Supplement: S1 Table — (DOCX) [file pone.0175113.s001.docx]

**S1 Table. P-distances among species of Chinese brown frogs based on *Cytb* (below the diagonal) and *COI* (above the diagonal).**

|  | (1) | (2) | (3) | (4) | (5) | (6) | (7) | (8) | (9) | (10) | (11) | (12) | (13) | (14) | (15) |
| --- | --- | --- | --- | --- | --- | --- | --- | --- | --- | --- | --- | --- | --- | --- | --- |
| (1) *R. dybowskii* |  | 0.134 | 0.129 | 0.131 | 0.163 | 0.172 | 0.156 | 0.172 | 0.177 | 0.183 | 0.177 | 0.168 | 0.154 | 0.154 | 0.125 |
| (2) *R. chensinensis* | 0.117 |  | 0.047 | 0.038 | 0.174 | 0.156 | 0.159 | 0.159 | 0.174 | 0.179 | 0.179 | 0.170 | 0.158 | 0.147 | 0.127 |
| (3) *R. huanrensis* | 0.119 | 0.070 |  | 0.048 | 0.172 | 0.149 | 0.154 | 0.161 | 0.170 | 0.179 | 0.183 | 0.165 | 0.165 | 0.142 | 0.122 |
| (4) *R. kukunoris* | 0.114 | 0.037 | 0.053 |  | 0.168 | 0.161 | 0.154 | 0.158 | 0.170 | 0.179 | 0.183 | 0.165 | 0.156 | 0.145 | 0.131 |
| (5) *R. kunyuensis* | 0.170 | 0.162 | 0.180 | 0.158 |  | 0.108 | 0.149 | 0.177 | 0.181 | 0.192 | 0.185 | 0.177 | 0.172 | 0.152 | 0.145 |
| (6) *R. amurensis* | 0.177 | 0.175 | 0.187 | 0.164 | 0.133 |  | 0.163 | 0.172 | 0.183 | 0.183 | 0.181 | 0.176 | 0.163 | 0.168 | 0.138 |
| (7) *R. chaochiaoensis* | 0.132 | 0.146 | 0.152 | 0.132 | 0.169 | 0.170 |  | 0.140 | 0.131 | 0.138 | 0.142 | 0.127 | 0.149 | 0.131 | 0.131 |
| (8) *R. jiemuxiensis* | 0.142 | 0.161 | 0.152 | 0.146 | 0.163 | 0.158 | 0.132 |  | 0.077 | 0.082 | 0.088 | 0.077 | 0.093 | 0.149 | 0.165 |
| (9) *R. culaiensis* | 0.143 | 0.148 | 0.165 | 0.148 | 0.150 | 0.170 | 0.111 | 0.086 |  | 0.032 | 0.027 | 0.072 | 0.081 | 0.147 | 0.156 |
| (10) *R. zhenhaiensis* | 0.141 | 0.139 | 0.155 | 0.151 | 0.156 | 0.168 | 0.115 | 0.084 | 0.024 |  | 0.041 | 0.077 | 0.077 | 0.142 | 0.165 |
| (11) *R. longicrus* | 0.136 | 0.152 | 0.173 | 0.151 | 0.166 | 0.181 | 0.126 | 0.100 | 0.035 | 0.042 |  | 0.086 | 0.091 | 0.152 | 0.167 |
| (12) *R. hanluica* | 0.143 | 0.148 | 0.163 | 0.150 | 0.150 | 0.145 | 0.107 | 0.084 | 0.077 | 0.082 | 0.088 |  | 0.077 | 0.131 | 0.147 |
| (13) *R. omeimontis* | 0.148 | 0.156 | 0.163 | 0.162 | 0.153 | 0.138 | 0.127 | 0.088 | 0.085 | 0.095 | 0.088 | 0.075 |  | 0.156 | 0.140 |
| (14) *R. japonica* | 0.121 | 0.136 | 0.138 | 0.130 | 0.179 | 0.164 | 0.126 | 0.121 | 0.106 | 0.106 | 0.135 | 0.104 | 0.115 |  | 0.136 |
| (15) *R. maoershanensis* | 0.155 | 0.165 | 0.167 | 0.160 | 0.177 | 0.167 | 0.155 | 0.159 | 0.158 | 0.156 | 0.171 | 0.153 | 0.172 | 0.132 |  |
