# Supplementary material for: Phylogeny and biogeography of South Chinese brown frogs (Ranidae, Anura)
Source: PLoS One. 2017 Apr 3;12(4):e0175113. doi: 10.1371/journal.pone.0175113 (PMC5378408; doi:10.1371/journal.pone.0175113)
Supplement: S2 Table — (DOCX) [file pone.0175113.s002.docx]

**S2 Table. P-distances among four main clades of Chinese brown frogs based on *Cytb* (below the diagonal) and *COI* (above the diagonal).**

|  | (1) | (2) | (3) | (4) |
| --- | --- | --- | --- | --- |
| (1) *R. amurensis* species group |  | 0.164 | 0.175 | 0.142 |
| (2) *R. chensinensis* species group | 0.172 |  | 0.168 | 0.127 |
| (3) *R. longicrus* species group | 0.160 | 0.150 |  | 0.153 |
| (4) *R. maoershanensis* | 0.173 | 0.163 | 0.161 |  |
