# Supplementary material for: Phylogeny and biogeography of South Chinese brown frogs (Ranidae, Anura)
Source: PLoS One. 2017 Apr 3;12(4):e0175113. doi: 10.1371/journal.pone.0175113 (PMC5378408; doi:10.1371/journal.pone.0175113)
Supplement: S1 Text — (DOCX) [file pone.0175113.s003.docx]

**S1 Text. Combination of same-species sequences from different specimens**

In this study, three species (*R. kukunoris, R. longicrus* and *R. japonica*) have Genbank sequences representing different loci that were taken from different specimens. We concatenated these sequences to represent the genetic characteristics of one species (Table 1). As our focus is on the phylogenetic relationships among major South Chinese brown frogs, intraspecific variation in each species should not affect our conclusions. We describe our method and decision to combine these sequences for each species below.

**1) Sequences with direct evidence**

The sequences of *R. kukunoris* and *R. japonica* were derived from different specimens. However, based on genetic evidence, we believe we avoided combining potentially cryptic species during concatenation.

- *Rana kukunoris*

The sequences of *R. kukunoris* came from three sources: Zhou et al. [[1](#_ENREF_1)], Yan et al. [[2](#_ENREF_2)], and Che et al. [[3](#_ENREF_3)] (Table 1). *Cytb* (JN984231; [[1](#_ENREF_1)]) and fragments of *12S* and *16S* (DQ289091 and DQ289116; [[3](#_ENREF_3)]) were all amplified from the specimen SCUM045101WD. The sequences of *Cytb* (JN984213; [[1](#_ENREF_1)]) and *COI* (JF939073; [[2](#_ENREF_2)]) were amplified from another specimen, CJ06102001. We calculated the uncorrected P-distance between the two fragments of *Cytb*, JN984231 (SCUM045101WD) and JN984213 (CJ06102001), as there was an overlap of 736 bp. The genetic distance was 0.1%, so we concatenated the sequences from the two specimens to represent the genetic characteristics of *R. kukunoris*.

- *Rana japonica*

The sequences of *R. japonica* originated from three sources: Sumida et al. [[4](#_ENREF_4)], Veith et al. [[5](#_ENREF_5)] and Yan et al. [[2](#_ENREF_2)] (Table 1). *Cytb* (AF077395; [[5](#_ENREF_5)]) and fragments of *12S* and *16S* (AB058858 and AB058876; [[4](#_ENREF_4)]) were amplified from the specimen jap.J^H^ that was sampled from Hiroshima, Japan. The sequences JF939138 and JF939101 from Yan et al. [[2](#_ENREF_2)], which include fragments of *Cytb* and *COI*, were amplified from another specimen from Japan, KIZYPX11775. We calculated the uncorrected P-distance between the two fragments of *Cytb,* AF077395 (jap.J^H^) and JF939138 (KIZYPX11775), as there was an overlap of 521 bp. The genetic distance was 0%, so we concatenated the sequences of the two specimens to represent the genetic characteristics of *R. japonica*.

**2) Sequences with indirect evidence**

- *Rana longicrus*

The sequences of *R. longicrus* were amplified from two different specimens: specimen long.T, with fragments of *12S* and *16S* (AB058863 and AB058881), from Sumida et al. [[4](#_ENREF_4)], and specimen KIZ15026, with fragments of *Cytb* and *COI* (JF939107 and JF939067), from Yan et al. [[2](#_ENREF_2)]. Although the two *R. longicrus* specimens were both from Taiwan, we could not directly concatenate the sequences, as there was no overlap. We concatenated the sequences based on indirect evidence through phylogenetic analyses. We reconstructed the ML phylogenetic tree of the *R. longicrus* specimen long.T, with the fragments of *12S* and *16S*, and specimen KIZ15026, with fragments of the *Cytb* and *COI* genes (S1 Fig). Although the phylogenetic positions of the two *R. longicrus* specimens were not identical, both showed a close relationship with *R. culaiensis* and *R. zhenhaiensis* (S1 Fig). The different tree topologies may be due to inherent differences in the loci. Therefore, we concatenated the sequences in our analyses.


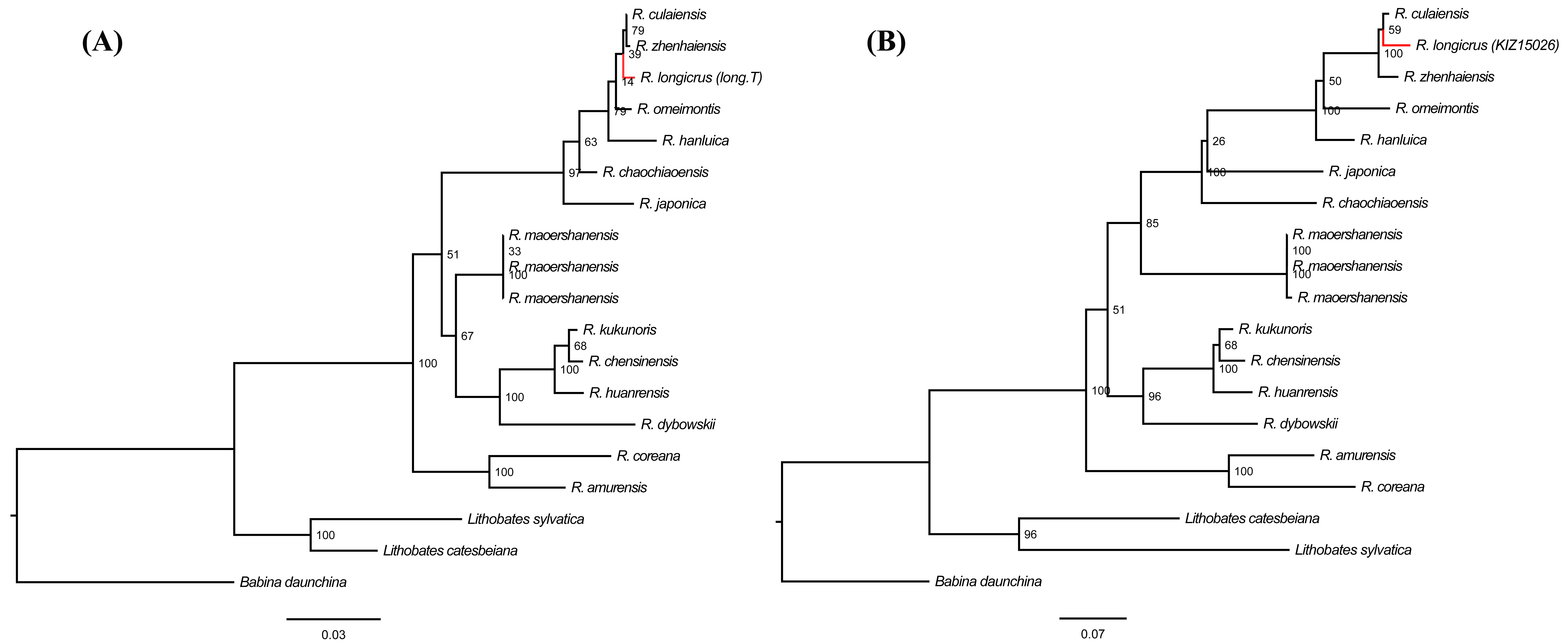


**S1 Figure. Partitioned best-scoring ML tree, inferred with 200 replications under the GTRCAT model.** (A) shows the ML tree base on *12S* and *16S* gene sequences, and (B) shows the ML tree bases on the *Cytb* and *COI* gene sequences.

**References**

1. Zhou W, Wen Y, Fu J, Xu Y, Jin J, Ding LI, et al. (2012) Speciation in the *Rana chensinensis* species complex and its relationship to the uplift of the Qinghai-Tibetan Plateau. Mol Ecol 21:960-973. doi: 10.1111/j.1365-294X.2011.05411.x.

2. Yan F, Jiang K, Chen H, Fang P, Jin J, Li Y, et al. (2011) Matrilineal History of the *Rana longicrus* Species Group ( *Rana* , Ranidae, Anura) and the Description of a New Species from Hunan, Southern China. Asian Herpetol Res 2:61-71. doi: 10.3724/sp.j.1245.2011.00061.

3. Che J, Pang J, Zhao E, Matsui M, Zhang Y (2007) Phylogenetic relationships of the Chinese brown frogs (Genus *Rana*) inferred from partial mitochondrial 12S and 16S rRNA gene sequences. Zool Sci 24:71-80.

4. Sumida M, Ueda H, Nishioka M (2003) Reproductive isolating mechanisms and molecular phylogenetic relationships among palearctic and oriental brown frogs. Zool Sci 20:567-580. doi: http://dx.doi.org/10.2108/zsj.20.567.

5. Veith M, Kosuch J, Vences M (2003) Climatic oscillations triggered post-Messinian speciation of Western Palearctic brown frogs (Amphibia, Ranidae). Mol Phylogenet Evol 26:310-327.
